# L’actinomycose thoracique multiple chez l’immunocompétent

**DOI:** 10.11604/pamj.2013.16.59.1753

**Published:** 2013-10-19

**Authors:** Yassine Msougar, Hicham Fenane, Mehdi Maidi, Abdellatif Benosman

**Affiliations:** 1 Service de Chirurgie Viscérale, CHU Mohamed VI, Marrakech, Maroc; 2 Service de chirurgie thoracique, CHU Ibn Sina, Rabat, Maroc

**Keywords:** Actinomycose, paroi thoracique, biopsie, actinomycosis, chest wall, biopsy

## Abstract

L′actinomycose est une affection bactérienne granulomateuse, suppurative, étendue et chronique provoquée par la bactérie anaérobique gram positif *Actinomyces israelii*. La localisation thoracique est rare, elle peut simuler une pathologie néoplasique ou une tuberculose. Il s’agit d’un patient de 54ans sans antécédents pathologiques, qui s’est présenté avec deux tuméfactions pariétales basithoarciques droites, l’une antérieure et l’autre postérieure s’accompagnant d’une altération de l’état général. L’examen clinique ainsi que le bilan radiologique ont montré deux masses de la paroi thoracique et une atteinte parenchymateuse basale droite. L’examen anatomopathologique de la biopsie de la masse antérieure a montré des foyers d’actinomycose permettant d’établir le diagnostic d’actinomycose thoraco-pulmonaire. Un bilan immunologique s’est révélé normal. Le patient est alors mis sous traitement antibiotique à base d’amoxicilline protégée avec bonne évolution clinique et radiologique. Le but de cette observation est de rappeler les aspects radio-clinique, histologiques, thérapeutiques et évolutifs ainsi que les difficultés diagnostiques de cette affection.

## Introduction

L'actinomycose est une affection ubiquitaire rare, due à une bactérie proche des champignons par son caractère filamenteux et ramifié. C’est une bactérie gram positif anaérobie, dont le diagnostic bactériologique est difficile et la présentation clinique peu spécifique. L'imagerie médicale, surtout par l'apport de la tomodensitométrie, permet d’évoquer le diagnostic dans les formes thoraciques [1]. Les localisations pariétales sont rare et sont dues à une extension soit par contigüité à partir des organes avoisinants, soit par voie hématogène.

## Patient et observation

**Figure 1:**
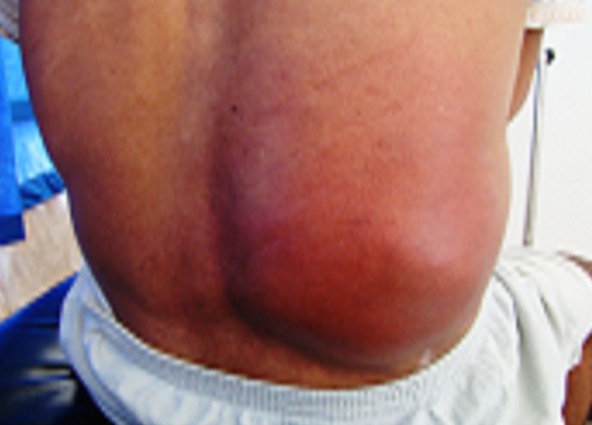
Masse basithoracique postérieure avec des signes inflammatoires en regard

**Figure 2:**
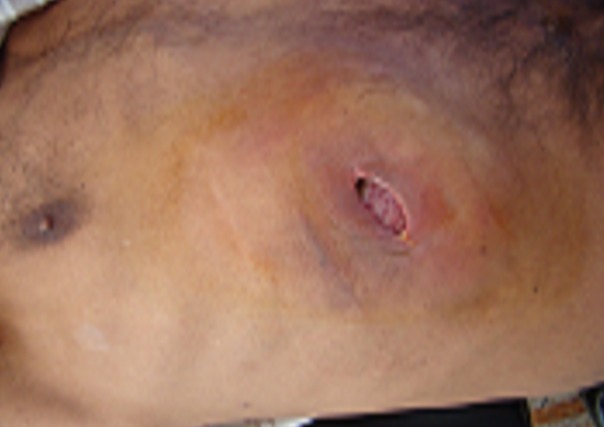
Masse basithoracique antérieure fistulisée a la peau (une biopsie a été réalisée)

Il s'agit d'un patient âgé de 40 ans, hospitalisé au service de chirurgie thoracique pour double tuméfactions de la paroi thoracique. En dehors d'un tabagisme, il ne présente pas d'antécédents médico-chirurgicaux particuliers, et notamment pas de notion de contage tuberculeux. Il présentait des douleurs thoraciques postérieures droites au niveau de sa cote flottante irradiant en antérieure, Il s'y associe deux tuméfactions inflammatoires augmentant rapidement de volume: l'une postérieure occupant tout le flanc droit (Figure 1), et l'autre antérieure en regard de la 6éme cote droite (Figure 2), le tout évoluant dans un contexte fébrile à 38°C, d'asthénie et d'altération de l′état général. L′examen clinique trouvait un mauvais état buccodentaire, une pâleur cutanéo-muqueuse, un blindage pariétal en regard des deux masses responsable d'une matité basale droite et d'une diminution des vibrations vocales. L′auscultation pulmonaire est normale et le reste de l'examen était sans anomalie; en particulier l'examen de l'abdomen et ORL était normal, les aires ganglionnaires étaient libres.

**Figure 3:**
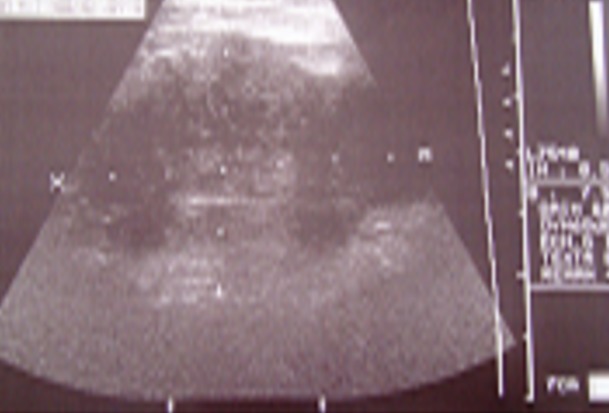
Echographie de la masse pariétale antérieure

Le bilan biologique montrait une vitesse de sédimentation accélérée à 80 mm à la première heure, une hyperleucocytose à 15600 éléments par mm3, à prédominance polynucléaire neutrophile (76%). La recherche de bacille de Koch dans les crachats faite successivement à trois reprises était négative, ainsi que l'intradermoréaction à la tuberculine. La sérologie HIV est négative, l'électrophorèse des protéines et le dosage des immunoglobulines se sont révélés normaux. La radiographie thoracique de face objectivait une opacité du cul de sac costo-diaphragmatique. La fibroscopie bronchique était normale. Une échographie de la masse pariétale antérieure avait montré : une masse pariétale et endothoracique basale droite tissulaire vascularisé au Doppler, de 6,6 cm d'axe transverse sur 4,4 cm d'axe antéropostérieur (Figure 3). Au scanner thoraco-abdominal, c'est une masse pariétale antérieure a la limite entre l'étage thoracique et abdominal et une autre postérieure a peine visible avec une atteinte du parenchyme pulmonaire (à noter que le scanner thoracique a été réalisé 18 jours avant que le patient ne soit admis au service de chirurgie thoracique), laissant suspecter une origine néoplasique. La biopsie réalisée chez le patient au niveau de la masse pariétale antérieure (Figure 2) a permis de prélever des fragments tissulaires friables avec issue du pus, l'étude anatomopathologique de la biopsie révélait le diagnostic d′actinomycose en montrant des grains *d′actinomyces*; la culture n'a pas été faite. Le patient fut mis sous traitement médical à base d′amoxicilline-acide clavulanique à raison de 3 grammes par jour par voie orale pendant 6 mois (le patient refusant l'hospitalisation) avec une bonne évolution clinique jugée sur l'apyrexie, une prise de poids et la diminution de volume de la tuméfaction.

## Discussion

### Epidémiologie

L'actinomycose est une infection rare, suppurative chronique qui a la capacité de s'étendre aux tissus adjacents, sans respect pour les barrières anatomiques normales [1]. Elle est caractérisée par sa tendance à former au sein des tissus des cavités à contenu purulent (granulome nécrosé), qui se fistulisent. Il existe souvent au sein de ces zones suppurées de petits grains jaunes caractéristiques, ou «granules sulfureux», de 2 à 3 mm de diamètre, correspondant à des micro-colonies d'actinomycoses, présentant un aspect en rayon de roue [2, 3]. Les germes responsables de l'actinomycose sont de la famille des actinomycètes. Le germe le plus important de ce groupe est Actinomyces, qui se compose de plusieurs espèces incluant A. Israelii, l'agent pathogène humain le plus habituel, découvert en 1981 par Israël [3, 4]. Cette pathologie est retrouvée dans le monde entier et ne présente pas de prédisposition raciale. Il existe, par contre, une nette prédominance masculine (3 hommes pour 2 femmes) [1]. Les enfants sont atteints de façon égale [5]. Ces organismes ne sont pas hautement virulents, mais sont retrouvés normalement dans l'oropharynx normal, particulièrement chez les personnes d'hygiène bucco- dentaire défectueuse. L'incidence dans les pays en voie de développement est élevée. Cette maladie est rare chez l'enfant parce que les caries dentaires et les périodontites sont rapidement suivies par la chute des dents [1]. L'atteinte cervico-faciale, plus exactement maxillaire, est la plus fréquente, et apparaît dans 55% des cas après extraction dentaire. Les atteintes abdomino-pelviennes représentent 20% des cas [6]. Les formes utérines associées à un dispositif intra-utérin sont en augmentation [7, 4]. La forme thoracique, qui représente 15 à 45% des cas [5–7], est secondaire, l'infection se faisant par voie descendante, bronchique ou médiastinale [2, 6]. Elle apparaît au décours d'un stress chirurgical, traumatique ou infectieux. L'infection thoracique fait suite à une aspiration de matériel infecté à partir de l'oropharynx. Ceci peut expliquer la prédominance basale classique de l'atteinte, ce qui a été le cas de notre patient qui présentait une très mauvaise hygiène bucco-dentaire [7, 8]. La lésion primaire touche le tissu péribronchique, les bronchioles et les alvéoles. Les organismes peuvent s'étendre du poumon à la plèvre, au médiastin, et à la paroi thoracique, sans tenir compte des barrières anatomiques. La raison est peu claire, mais peut être en rapport avec l'activité protéolytique de la bactérie. Une dissémination hématogène peut se faire à la suite de l'infection thoracique. Inversement, l'atteinte thoracique peut se faire par extension directe à partir du cou, de l'Œsophage, de l'abdomen ou du rétropéritoine [5, 9]).

### Clinique

Les patients présentent une ou plusieurs masses pariétales, qui augmentent progressivement de volume, fébricule et perte de poids (18), simulant une pathologie néoplasique, avec ou sans signes respiratoires: toux, expectorations, douleurs thoraciques, hémoptysies ou dyspnée. En l'absence de traitement, l'évolution de l'atteinte pariétale peut se faire vers l'abcédation et la fistulisation à la peau.

### Imagerie

Les éléments retrouvés à la radiographie standard dépendent de la chronicité de la maladie. On note une prédominance de l'atteinte périphérique du lobe inférieur, reflétant le rôle de l'aspiration dans la pathogénie de cette maladie. La tomodensitométrie permet une meilleure caractérisation des lésions. Ainsi, l'actinomycose thoracique se caractérise en TDM, par un syndrome de condensation pulmonaire avec épaississement pleural adjacent. Elle montre une cavitation ou la présence de zones de basse densité témoignant de la formation d'abcès [2]. Un envahissement endobronchique a déjà été décrit, simulant un cancer [7]. Toutefois, des cas de culture positive d'actinomycose sur des masses parenchymateuses de cancer bronchique ont également été rapports [8]. L'invasion des structures médiastinales a rarement été décrite, ainsi que l'extension trans-diaphragmatique au niveau de l'abdomen et la possibilité d'une fistule intercostale [10]. L'extension pleurale et pariétale est fréquente [9, 10]. La sécrétion d'enzymes protéolytiques par actinomycoses pourrait expliquer ce franchissement des barrières anatomiques. L'épaississement pleural est typiquement fin et régulier (< 1 cm d'épaisseur), similaire à celui noté chez notre patient, témoignant de son origine inflammatoire [4]. Les épanchements pleuraux et les adénopathies hilaires sont également communs [3]. L'atteinte pariétale se traduit par une masse ou une infiltration tissulaire. Les appositions périostées costales peuvent prendre un aspect en vague et sont très évocatrices du diagnostic [5, 6, 9]. Bates considère même que l'existence d'appositions périostées costales en l'absence d'empyème est un signe pathognomonique d'actinomycose. Dans le cas de notre patient, nous n'avons pas retrouvé d'appositions périostées, mais plutôt des lésions lytiques. Ceci aurait pu poser un problème de diagnostic différentiel avec une pathologie tumorale maligne. Mais l'aspect diffus, non nodulaire de l'épaississement des parties molles adjacentes, nous a plus orienté vers une pathologie inflammatoire notamment une tuberculose pariétale, une cryptococcose ou une blastomycose [10].

### Diagnostic bactériologique et/ou histologique

Le diagnostic de certitude se fait par l'isolement du germe dans le pus de ponction ou d'écoulement spontané et par sa mise en culture [1, 5]). Il est souvent histologique, reposant sur l'étude anatomopathologique de la biopsie de la tuméfaction, et confirmés par la mise en évidence de lésions en forme de grains jaunes dans les tissus «sulfure granules » donnant un aspect en rayon de roue quasi pathognomonique [3, 5, 6]. Le diagnostic reste difficile, et se fait habituellement avec un retard pouvant aller de un mois à deux ans, non seulement à cause des difficultés liées à l'obtention de prélèvements fiables, bactériologiques ou histologiques, mais aussi à cause de la sensibilité habituelle du germe aux antibiotiques usuels qui peuvent négativer transitoirement le tableau clinique.

### Pronostic

Le pronostic de l'actinomycose thoracique est moins bon que dans les autres localisations. En l'absence de traitement, la maladie évolue vers la dissémination métastatique (hépatique, cérébrale) et surtout l'envahissement de la paroi thoracique comme est le cas dans notre observation. Le décès survient dans 60 à 80% des cas [9, 10]. Le traitement antibiotique a transformé cette évolution, puisque la guérison est obtenue dans 70 à 80% des cas [3, 5, 9]. Les actinomyces sont des germes très sensibles aux antibiotiques. Le traitement de référence est la Pénicilline G par voie parentérale à fortes doses (10 à 20 millions/jour) durant 2 à 6 semaines, relayée par la voie orale (2 à 4 grammes par jour pendant plusieurs mois), en moyenne 3 à 12 mois car les rechutes sont possibles [9, 10]. D'autres antibiotiques ont été utilisés (sulfamides, cyclines, ampicilline, macrolides). Les céphalosporines orales semblent généralement peu efficaces sur les actinomyces. Dans tous les cas, l'antibiothérapie doit être systématiquement associée à un traitement stomatologique soigneux [3, 5, 7]. Le traitement chirurgical peut être indiqué pour le drainage des grands abcès, empyèmes [7] et le débridement des trajets fistuleux.

## Conclusion

L'actinomycose thoracique est une pathologie infectieuse rare, évoquée devant tout syndrome thoracique sombre. Le tableau clinico-radiologique est trompeur, pouvant simuler une pathologie tumorale ou tuberculeuse d'ou la nécessité de refaire des biopsies à la recherche des caractères histologiques de l'actinomyces.

## References

[1] de la Espina MA, Lopez-Menendez C, Ruiz-Martinez R, Molino-Trinidad C (2001). Pulmonary actinomycosis with thoracic soft tissue mass: a rare onset form. Eur J Radiol.

[2] Kwong JS, Muller NL, Godwin JD et coll (1992). Thoracic Actinomycosis: CT Findings in Eight Patients. Radiology.

[3] Allen HA, Scatarige JC, Kim MH (1987). Actinomycosis: CT findings in six patients. AJR Am J Roentgenol.

[4] Ariel I, Breuer R, Kamal NS, Ben-Dov I, Mogel P, Rosenmann E (1991). Endobronchial actinomycosis simulating bronchogenic carcinoma - Diagnosis by bronchial biopsy. Chest.

[5] Balikin JP, CHeng TH, Costello P, Herman PG (1978). Pulmonary actinomycosis: a report of three cases. Radiology.

[6] Bates M, Cruiskshank G (1957). Thoracic Actinomycosis. Thorax.

[7] Afif H, Aichane A (1999). Opacité pulmonaire inhomogène pseudo-tumorale. Rev Mal Respir.

[8] Choudat D, De Gramont A, Bellamy J et coll (1980). Actinomycose pulmonaire greffée sur des lésions tuberculeuses anciennes: A propos d'une observation. Rev Fr Mal Respir.

[9] Bennhoff DF (1984). Actinomycosis: diagnostic and therapeutic considerations and a review of 32 cases. Laryngoscope.

[10] Brauner M, Hamidou A, Goldlust D (1999). Syndrome cavitaire. Encycl Méd Chir (Elsevier, Paris), Radiodiagnostic — Cœur-Poumon..

